# Mimicking titration experiments with MD simulations: A protocol for the investigation of pH-dependent effects on proteins

**DOI:** 10.1038/srep22523

**Published:** 2016-03-03

**Authors:** Eileen Socher, Heinrich Sticht

**Affiliations:** 1 Division of Bioinformatics, Institute of Biochemistry, Friedrich-Alexander-University Erlangen-Nürnberg (FAU), Fahrstraße 17, 91054 Erlangen, Germany

## Abstract

Protein structure and function are highly dependent on the environmental pH. However, the temporal or spatial resolution of experimental approaches hampers direct observation of pH-induced conformational changes at the atomic level. Molecular dynamics (MD) simulation strategies (e.g. constant pH MD) have been developed to bridge this gap. However, one frequent problem is the sampling of unrealistic conformations, which may also lead to poor pK_a_ predictions. To address this problem, we have developed and benchmarked the pH-titration MD (pHtMD) approach, which is inspired by wet-lab titration experiments. We give several examples how the pHtMD protocol can be applied for pK_a_ calculation including peptide systems, *Staphylococcus nuclease* (SNase), and the chaperone HdeA. For HdeA, pHtMD is also capable of monitoring pH-dependent dimer dissociation in accordance with experiments. We conclude that pHtMD represents a versatile tool for pK_a_ value calculation and simulation of pH-dependent effects in proteins.

Solution pH can have a drastic effect on protein structure and function, which has been exploited by nature to trigger a large variety of physiological processes. For example, some bacteria are able to survive the acidic conditions in the stomach of their host by using acid-activated chaperones which protect substrate proteins upon binding^1^. In viruses, some of the fusion proteins that mediate cell entry have been described to act pH-dependently^2^,^3^. Other proteins in vertebrates undergo pH changes during their maturation on the way through the endoplasmic reticulum and the Golgi apparatus^4^. In plants, the simultaneous closure of water channels has been observed as a response to changing pH values during flooding^5^.

On a molecular level, changes in the pH value affect the protonation state of several types of amino acids, including aspartate, glutamate, histidine, lysine, cysteine, and tyrosine. The addition or removal of a proton always changes the charge of the respective amino acid side chain, thereby affecting the charge distribution within the protein, which may lead to conformational changes. For instance, these structural alterations can trigger changes in protein activity, ligand binding properties, or the oligomerization state.

However, due to the temporal or spatial resolution of experimental approaches, it is extremely difficult to observe pH-induced conformational changes in proteins directly at the atomic level. Also the generation of structural data at different pH values, for instance with X-ray crystallography or NMR spectroscopy, underlies different restrictions and is technically very demanding. To mention only a few general limitations, proteins mostly do not crystallize at very different pH values and NMR spectroscopy is limited to small proteins.

At this point, molecular dynamics (MD) simulations, which start from experimentally determined structures, can help investigate the effect of pH changes on an atomic level and on picosecond to microsecond time scales. One hallmark of conventional MD simulations is the fact that an initially assigned protonation state cannot be changed during the simulation. This “constant protonation” approach results in some drawbacks for studying pH-dependent effects^6^: (1) Assigning the right protonation states for the titratable groups in the protein requires knowledge of their pK_a_ values, (2) if any of these pK_a_ values are near the solvent pH there may be no single protonation state that adequately represents the ensemble of protonation states appropriate at that pH, and (3) the invariable protonation states decouple the dynamic dependence of pK_a_ and protonation state on conformation.

To avoid these problems, the constant pH molecular dynamics (CpHMD) approach was developed^6^,^7^. One widespread implementation, for example in the AMBER software suite, performs Monte Carlo sampling of the Boltzmann distribution of protonation states interspersed in the molecular dynamics simulation^8^. Thereby, the solution pH is set as an external variable determining the distribution of the different protonation states, which are modeled by different charge sets^8^.

CpHMD has become a popular method to study the pH-dependence of protein^9^ and peptide^10^ structures or to calculate the pK_a_ values of titratable residues^6^,^11^. However, a comparison between calculated and experimentally determined pK_a_ values frequently revealed significant differences indicating that unrealistic protein conformations are sampled^11^,^12^. Recent approaches to reduce this problem are constant pH replica exchange molecular dynamics (pH-REMD) simulations^13^,^14^ and the explicit consideration of the solvent^12^,^15^.

As an alternative approach, we have devised a modified procedure, which is inspired by wet-lab titration experiments. This pH-titration MD (pHtMD) relies on the overall concept of CpHMD, but performs a consecutive series of MD simulations with small pH changes, which allows a smooth adaption of the structure to the solvent pH (Fig. 1).

The rationale for suggesting this titration concept was the following: Conventional CpHMD usually runs a set of simulations at different pH values that are fixed at the beginning of each simulation and may differ significantly from the pH at which the structure was determined (e.g. pH 3 simulation using a pH 8 structure as a template). CpHMD thus requires a rapid adaptation of the structure to different pH values, which may cause the sampling of unrealistic conformations thereby producing inaccurate pK_a_ values.

To address this problem, we have developed and benchmarked the pHtMD approach as described in the present manuscript. First, we demonstrate that our approach is capable of accurately reproducing the pK_a_ values of small model peptides. Application of the approach to the staphylococcal nuclease (SNase) mutant Δ+PHS demonstrates that the pHtMD method performs better than conventional CpHMD methods for the prediction of pK_a_ values for this system. For the dimeric HdeA protein, pHtMD predicts pK_a_ values with a similar accuracy as recent pH-REMD simulations and is also capable of monitoring dimer dissociation at low pH values in accordance with experiments. We conclude that pHtMD represents a versatile tool for the calculation of pK_a_ values and the simulation of pH-dependent motions in proteins.

## Results

### Prediction of pK_a_ values for model peptides

**Figure 2:**
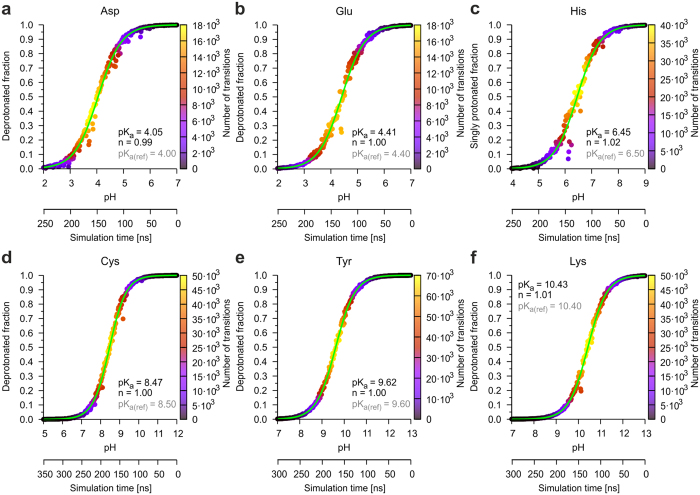
Titration curves for side chains in the Ace-X-Nme model compounds. (**a**–**f**) Observed titration curves for the amino acid side chains in the Ace-X-Nme model compounds (X = Asp, Glu, His, Cys, Tyr or Lys) from MD simulations in which the pH was gradually lowered over time. The measured deprotonated fraction or the singly protonated fraction in the case of histidine is color-coded according to the number of transitions. As expected, the highest number of transitions can be observed near the pK_a_ value. Additionally, the simulation time is shown as a second x-axis for orientation. The fitted curve, used for the calculation of the pK_a_ value and the Hill coefficient n, is shown as a green line. Reference pK_a_ values are given as gray text (reference values for Asp, Glu, Tyr, Lys taken from Bashford *et al.*^39^; value for His taken from McNutt *et al.*^40^; and value for Cys taken from Stryer^41^).

The energy function for titratable amino acids in AMBER has been designed in that fashion that the experimentally observed pK_a_ values are reproduced, when simulations are performed according to the standard CpHMD approach implemented in AMBER. Therefore, the first aim of our work was to ensure that simulations according to the pHtMD protocol are still consistent with the AMBER parametrization and do not result in unrealistic pK_a_ values. To address this point, Ace-X-Nme model peptides were built for Asp, Glu, His, Cys, Tyr, and Lys residues. To eliminate effects on the amino acid side chain pK_a_ values by charged termini, the terminal ends were capped with N-terminal acetyl (ACE) and C-terminal N-methylamine groups (NME). The simulations of these model compounds started with deprotonated states at higher pH values and were slowly decreased, so that each titratable side chain became protonated. By plotting the deprotonated fraction over the pH range, titration curves can be fitted (Fig. 2a–f). The difference between the calculated pK_a_ values and the reference pK_a_ values of the side chains (implemented in AMBER) is maximally 0.05. The Hill coefficients are between 0.99 and 1.02. All in all, the findings for the model compounds are in good agreement with the expected results.

**Figure 3:**
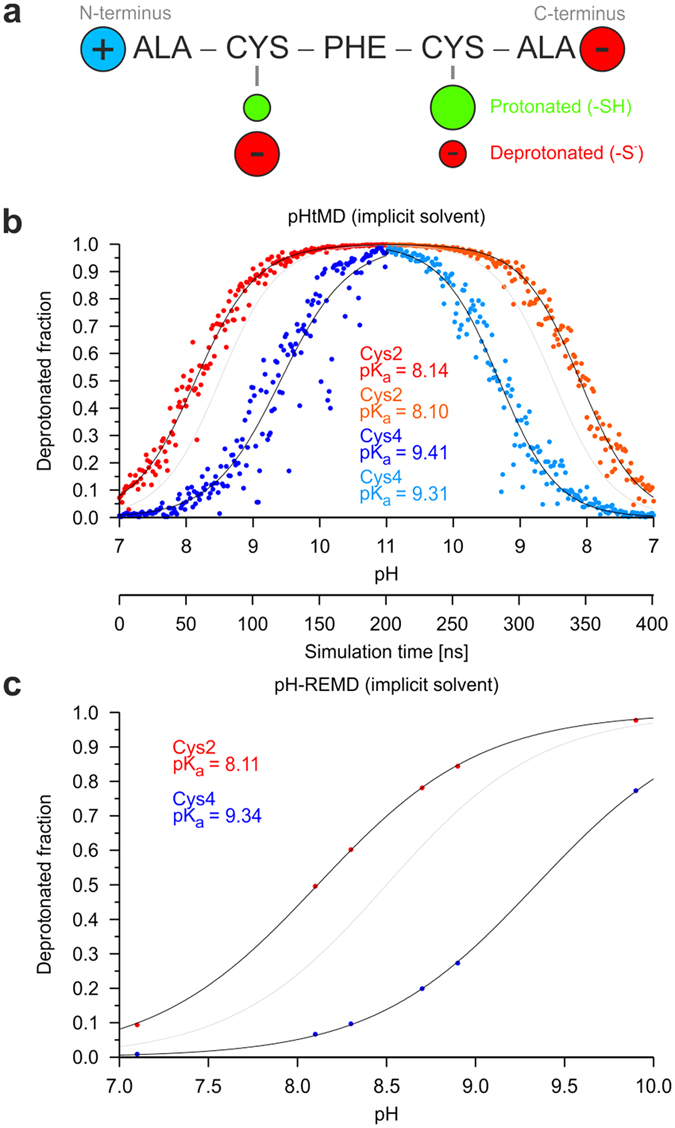
Titration curves for the cysteines in the ACFCA pentapeptide. (**a**) In the ACFCA pentapeptide, Cys2 prefers the deprotonated, negatively charged state due to electrostatic interactions with the positively charged N-terminus. Cys4, in contrast, prefers the protonated, uncharged state due to electrostatic interactions with the negatively charged C-terminus. (**b,c**) Titration curves for the cysteines in ACFCA obtained from (**b**) a pHtMD simulation starting at pH = 7 (During the pHtMD simulation the solution pH was first increased to 11 and then again decreased to 7.) (**c**) a pH-REMD simulation both with implicit solvent (six replicas; each run for 10 ns). For comparison, the titration curve for the Ace-Cys-Nme is shown as a gray line.

The pentapeptide Ala-Cys-Phe-Cys-Ala (ACFCA) had been designed as a model system by Swails *et al.*^12^ for the development of pH replica exchange MD (pH-REMD) simulation methods. The peptide contains two charged termini as well as two titratable cysteines. Due to the charge, the termini interact electrostatically with the neighboring cysteines, thereby influencing their pK_a_ values (Fig. 3a).

The ACFCA pentapeptide was simulated according to the pHtMD strategy described in Methods. First, the peptide was titrated from pH 7 to pH 11 within 200 ns (Fig. 3b, left half). As expected, the titration curves of the cysteines are shifted to the left or right with respect to the titration curve of the Ace-Cys-Nme model compound, due to the influence of the charged termini of the pentapeptide. The titration curve of Cys2 is shifted to a lower pK_a_ value of 8.14 because of electrostatic interactions with the positively charged N-terminus, whereas the pK_a_ value of Cys4 is increased to 9.41 due to the vicinity of the negatively charged C-terminus (Fig. 3b). In a second step we checked for hysteresis by titrating the peptides back from pH 11 to pH 7 (Fig. 3b, right half). The pK_a_ values obtained from both titrations for the cysteines differ by ≤0.1 units. To allow a comparison to other approaches, a pH-REMD simulation in implicit solvent was performed using a total of six replicas at the following pH values: 7.1, 8.1, 8.3, 8.7, 8.9, and 9.9 (Fig. 3c). These pH values were adapted from the pH-REMD study by Swails *et al.*^12^ that was performed for the same peptide in explicit solvent.

There is a good agreement between both simulation strategies and the determined pK_a_ values differ by less than 0.1 units. In addition, the values obtained are also close to those reported by Swails *et al.* for pH-REMD simulations in explicit solvent (cysteine pK_a_ values of 8.16 and 9.37, respectively)^12^. An additional feature of pHtMD is the larger number of data points generated compared to conventional CpHMD or pH-REMD simulations (see Fig. 3b vs. 3c). This makes curve fitting less dependent on the inaccuracies of individual data points.

Thus we conclude that for small model compounds the performance of pHtMD is similar to that of other state-of-the-art methods. The next simulations investigated whether pHtMD also gives good pK_a_ predictions for globular proteins.

### Prediction of pK_a_ values for globular proteins

**Figure 4:**
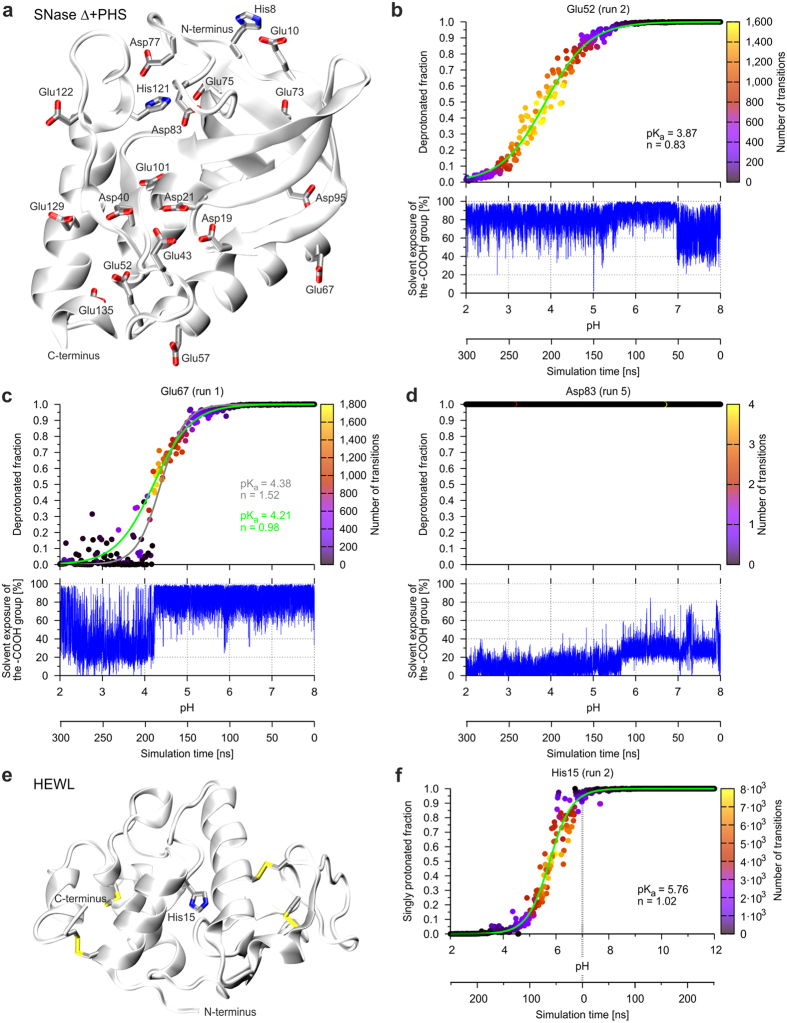
Titration of acidic side chains in the staphylococcal nuclease mutant Δ+PHS and of the His15 side chain in HEWL. (**a**) Overall structure of the SNase mutant Δ+PHS. The side chains of the Asp, Glu, and His residues are shown as sticks. (**b**) Titration curve of Glu52 of the SNase mutant Δ+PHS. Additionally, the SASA of the carboxyl group is plotted over pH/simulation time showing that the carboxyl group is solvent accessible. (**c**) Below pH 4, the carboxyl group of Glu67 is not as solvent accessible as at the beginning of the simulation. This has a noticeable influence on the titration curve below pH 4 and the fitted curve (gray line). Excluding the data at which the carboxyl group is buried from the fitting procedure (see Methods) leads to the new and better fitted curve (green line). (**d**) The carboxylic group of the side chain of Asp83 is buried in the whole simulation, so that it does not titrate between pH 8 and pH 2 and it is impossible to calculate a pK_a_ value. (**e**) Overall structure of HEWL. For orientation, disulfide bridges are highlighted in yellow. The side chain of His15 is shown as a stick representation. (**f**) Titration curve of the side chain of His15 obtained from the combination of two pHtMD simulations either covering pH 7 to 2 or pH 7 to 12 (delimited by a gray dashed line in the plot). The fitted curve (green line) was used for the calculation of the pK_a_ value and the Hill coefficient n.

The staphylococcal nuclease (SNase) is a highly charged protein^11^, which contains 149 residues and carries no disulfide bonds. To test our simulation protocol, we investigated the SNase Δ+PHS mutant in the pH range between 8 and 2. This mutant represents a hyperstable, acid-resistant SNase with five substitutions (G50F, V51N, P117G, H124L, and S128A) and a deletion of residues 44–49^16^ (Fig. 4a). For the simulations, all Asp, Glu, His, Tyr, and Lys residues of the crystal structure were set as titrating residues. Representative titration curves are shown in Fig. 4b–d and the number of transitions observed as well as the solvent exposure of the respective group is shown.

Glu52 (Fig. 4b) is a residue that is highly solvent exposed between pH 2 and 7 and a sufficiently large number of transitions between the different protonation states is observed around the pK_a_ value thus providing a good basis for the fitting of the titration curve and calculation of the pK_a_ value. In contrast to Glu52, Glu67 (Fig. 4c) is significantly buried at pH < 4, which results in a low number of transitions observed. Consequently, a reliable calculation of the deprotonated fraction is not possible for the respective data points, which also hampers pK_a_ value calculation. The inclusion of all data points for curve fitting yields a pK_a_ value of 4.38 for Glu67 (gray line in Fig. 4c). Excluding the data at which the carboxyl group is exposed <30% from the fitting procedure (see Methods) leads to the new fitted curve (green line). The resulting pK_a_ value of 4.21 is now closer to the experimentally determined pK_a_ value of 3.76^16^.

Glu67 is also a good example to demonstrate that the correlation between experiment and prediction can be improved by running multiple independent pHtMD experiments. For SNase Δ+PHS a total of six simulations were performed and resulted in pK_a_ values of 4.21, 3.85, 3.78, 3.52, 3.64, and 4.05. The average value of 3.84 (Table 1) is in better agreement with the experimental pK_a_ value of 3.76 than the results from the individual simulations.

The exclusion of data points collected for significantly buried side chain conformations proved to be beneficial for those residues that are only buried for part of the simulation time. However, this approach cannot be applied to residues that are buried for most of the simulation time. This is, for example, the case for the three aspartates of the active site (Asp19, Asp21, Asp40) and for Asp83 (Fig. 4d). Consequently, no pK_a_ value was calculated for these residues. The problem that no reliable pK_a_ values can be obtained for buried residues showing a low number of transitions between different protonation states has already been noted in previous CpHMD^11^ studies. The experimental study conducted by Castañeda *et al.* showed that neither Asp77 nor Asp83 titrated between pH 2 and pH 9 and that the oxygen atoms of these residues were less than 10% solvent-accessible^16^. All pK_a_ values obtained from the pHtMD approach are summarized in Table 1 and compared to experimental and alternative computational approaches. Inspection of the average deviation between experimental and predicted pK_a_ values reveals that our approach performs better for SNase Δ+PHS than a previous conventional CpHMD simulation^11^ or PROPKA pK_a_ predictions based on the static crystal structure.

The lack of consideration of the protein dynamics could also be the reason why the predicted pK_a_ values of PROPKA differ sometimes more than one pH unit from the experimental values (e.g Glu73, Glu101, and His121). For these residues, the pHtMD simulation protocol predicted more precise pK_a_ values (pK_a_ differences: <0.6 pH units). The improvement compared to the conventional CpHMD simulation most likely results from a more accurate sampling of the conformational space, which was already identified as a key prerequisite for the prediction of reliable pK_a_ values by Williams *et al.*^11^.

In our study of SNase above, pK_a_ values were obtained from single pHtMD experiments that covered a pH range from 8 to 2. Next, we investigated whether two pHtMD simulations may be combined to cover a broader pH range. As an example we selected hen egg-white lysozyme (HEWL), which contains a single histidine residue (Fig. 4e). Two pHtMD simulations were performed either covering a pH range from 7 to 2 or from 7 to 12. The rationale for choosing pH 7 was to start at a pH value for which an experimentally determined protein structure is available as starting point of the simulations. Figure 4f shows that the two resulting datasets can be combined to cover the entire pH range from 2 to 12.

The strategy above was repeated four times and resulted in an average pK_a_ value of 5.69, which is close to the pK_a_ values found for His15 in experiments: 5.68–5.74^17^ or 5.5 ± 0.2^18^. The pHtMD prediction is also closer to the experimental values than predictions from PROPKA (pK_a_ = 6.31) or from conventional CpHMD simulations (pK_a_ = 6.45^6^).

### Prediction of pH-dependent motions in globular proteins

HdeA, the small acid stress chaperone, is important for *Escherichia coli* to survive in the acid environment of the host stomach because it binds other periplasmic proteins and prevents their aggregation. At neutral pH, HdeA forms homodimers, which dissociate into disordered monomers when the pH decreases^19^,^20^. We have selected HdeA as a model system to investigate whether pHtMD can also monitor larger-scale structural changes in addition to predicting pK_a_ values. The pHtMD simulations started with the HdeA dimer, for which a crystal structure is available, and simulated a slow decrease of the pH from 7 to 2 within 250 ns.

The pK_a_ values of all aspartates and glutamates were calculated and are summarized in Table 2. The average deviation of 0.27 from the experimentally determined pK_a_ values is smaller than that resulting from PROPKA predictions (average deviation =0.52). This better performance of pHtMD indicates the requirement of an accurate conformational sampling, which is particularly relevant for proteins such as HdeA that undergo large conformational rearrangements upon pH changes.

As an alternative approach to obtain more realistic pK_a_ values, pH-REMD has been recently performed for HdeA by Ahlstrom *et al.*^21^. The results from this approach are also given in Table 2 for comparison. Depending on the oligomerization state chosen for simulation (monomer vs. dimer) pH-REMD results in average deviations of 0.16 and 0.33 pH units from the experimental pK_a_ values; this is in the same range as for our pHtMD protocol.

**Figure 5:**
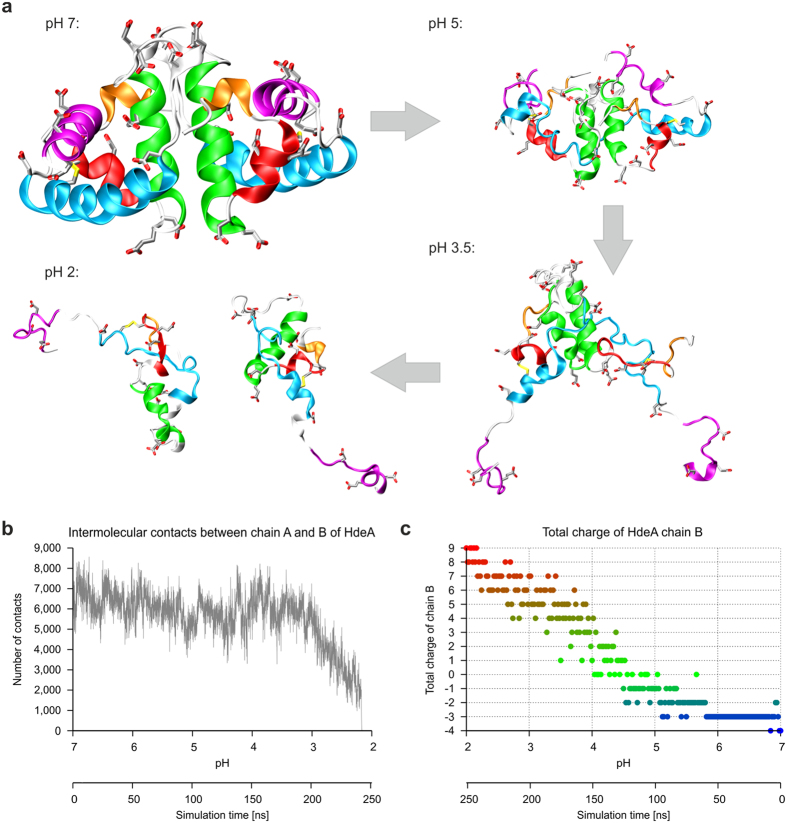
pH-dependent dissociation of the HdeA dimer of *Escherichia coli*. (**a**) Snapshots from the pHtMD simulation showing representative structures for pH 7.0, 5.0, 3.5 and 2.0. All titrating aspartates and glutamates are shown as sticks. The disulfide bond is highlighted in yellow. (**b**) Number of intermolecular contacts between monomer A and monomer B over the simulation. (**c**) Overall charge of the HdeA monomer B as a function of pH. The negatively charged monomer from the simulation start becomes neutral around pH 4 and is positively charged at the end of the simulation.

**Figure 6:**
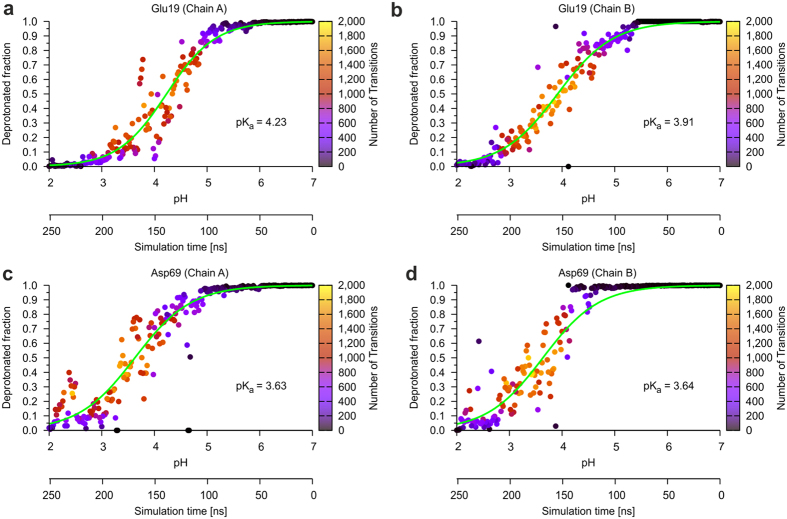
Titration curves of acidic residues. (**a**,**b**) Glu19 and (**c**,**d**) Asp69 of HdeA. The residues were analyzed separately for each subunit of the protein. Curves were fitted according to the procedure described in methods and the number of transitions is shown as a bar on the right of each diagram.

We also investigated whether pHtMD does not only allow to calculate pK_a_ values but also is able to monitor pH-dependent HdeA dissociation. In fact, it was possible to observe the dissociation process and the transition from a well-folded dimeric structure into disordered monomers (Fig. 5a). Unfolding and subsequent dissociation occurs in the pH range from 3.5 to 2 (Fig. 5a), which is also reflected by the gradual decrease of intermolecular contacts (Fig. 5b). At pH 2.2 all intermolecular contacts are lost. Since unfolding and dissociation occur over a rather broad pH range, they are covered by >50 ns of the simulation time. This is also reflected in the gradual changes of the protonation states of the titrating groups (Fig. 6), which allow curve fitting and calculations of the pK_a_ values. The structural properties of HdeA monitored during the pHtMD simulation are also in line with NMR spectroscopic data indicating that HdeA remains dimeric from pH 6 down to pH 3^22^ and that an unfolded monomeric conformation is present at pH 2.5^20^.

The analysis of the pHtMD simulation also allows extracting additional properties of the system such as the overall charge as a function of the pH (Fig. 5c). In the respective plot, the overall charge is calculated from the protonation state of the ionizable groups after every nanosecond of simulation resulting in 250 data points. The result approximates the pH range in which a particular net charge is observed. The respective plot can be directly calculated based on the protonation states observed and does not rely on an interpretation of pK_a_ values that may be difficult to obtain for buried side chains (see Fig. 4c,d above for examples).

## Discussion

In the present work, we have devised the pHtMD protocol, which is based on the CpHMD approach, to calculate pK_a_ values and to simulate pH-dependent effects on proteins. The concept of pHtMD aims to perform a consecutive series of MD simulations with small pH changes in a defined direction to allow a smooth adaption of the structure to the solvent pH. This differs from the traditional CpHMD approach, in which a set of simulations is run at different pH values, which may differ significantly from the pH at which the structure was determined (e.g. pH 2 simulation using a pH 8 structure as a template). Consequently, the structure needs to abruptly adapt to the lower pH, which may cause the sampling of unrealistic conformations thus producing inaccurate pK_a_ values.

pHtMD was applied to model peptides and globular proteins to assess the potential range of applications: SNase Δ+PHS represents a protein system that exhibits strongly shifted pK_a_ values, and HdeA is a protein that undergoes subunit dissociation at low pH. For the SNase and HdeA systems, the pK_a_ of the titratable groups was predicted with an average deviation of 0.45 and 0.27, respectively (Tables 1 and 2). For HdeA, this accuracy is within the range observed for recent pK_a_ predictions using the pH-REMD strategy^21^. In addition, the average errors of pHtMD are also within the estimated uncertainty of 0.5 pH units for pK_a_ values determined by NMR experimental techniques^18^. As one key feature of pHtMD, a large pH range can be scanned within one simulation and no prior pK_a_ value estimations of individual residues is required for the setup. Similar to other CpHMD-based protocols, pHtMD has problems to predict reliable pK_a_ values for highly buried residues that do not titrate within a physiological pH range. However, due to their strongly shifted pK_a_ values, these residues do not function as triggers of physiologically relevant pH-induced conformational changes. Therefore, the inability to calculate pK_a_ values of those residues does not critically affect the applicability of the pHtMD approach to investigate pH-dependent effects on protein structure that occur in a physiological pH range.

For HdeA it was possible to monitor the dissociation process and the pH range for dissociation is in close agreement with the experiment^19^,^22^. As one advantage, the pHtMD method does not require *a priori* experimental knowledge of whether dissociation occurs and there is no need for an explicit consideration of dimeric and monomeric states in separate simulations. Instead, the dissociation process can be monitored within one single pHtMD run and does not require including additional assumptions for the setup of the simulation.

Similar to other simulation protocols, several repetitions of each pHtMD simulation allows increasing accuracy of the results, which is particularly important for systems with partially buried titratable residues such as SNase Δ+PHS. Multiple simulations reduce the risk that an unrealistic conformational sampling within a single simulation might result in a wrong prediction of pK_a_ values.

For protein systems that exhibit large conformational changes within a narrow pH-range, the currently used rate of pH change (0.02 units per nanosecond) might be too high. We therefore suggest to use smaller rates of change if there is evidence for such motions, e.g. from unusual titration curves or from experimental data.

We always suggest to start the pHtMD at a physiological pH value for which an experimental structure is available. Starting at extreme pH values may work for peptides, which exhibit a nonglobular structure that is rather independent of pH. However, it may fail for globular proteins that frequently undergo unfolding under such conditions. This can be illustrated by considering HdeA, which exists in a monomeric state at pH 2. A pHtMD experiment starting at pH 2 would require to simulate the association of two unfolded monomers into a correctly folded dimer, which is not yet feasible by MD simulations.

A further increase in the accuracy of pHtMD simulations may be achieved by an explicit consideration of the solvent in the future. Explicit solvent has been recently used in conjunction with pH-REMD and resulted in an improved prediction of pK_a_ values^12^,^15^. Simulations in explicit solvent should also allow studying the role of ions, especially of those ions bound to the protein, or of a membrane environment. The concept of pHtMD is appropriate for a combination with explicit solvent; however, extensive benchmarking will be required to determine suitable simulation parameters.

Furthermore, we assume that the principle of our simulation approach can be easily transferred to other simulation programs and is not restricted to the Amber software suite, because pHtMD is based on the well-established CpHMD approach^6^,^23^,^24^,^25^, which is also implemented in other molecular dynamics software packages such as GROMACS^26^ or CHARMM^27^,^28^.

## Methods

### Starting structure generation

The structures of the Ace-X-Nme model compounds (X = Asp, Glu, His, Cys, Tyr, Lys) and the Ala-Cys-Phe-Cys-Ala (ACFCA) model peptide were built using the *tleap* module of Amber 12^8^.

The structures of the proteins investigated were retrieved from the protein data bank^29^: SNase Δ+PHS (3BDC^16^), HEWL (1AKI^30^), and dimeric HdeA (1BG8^31^). If the terminal residues did not represent the natural termini, an acetyl group (ACE) was added at the N-terminus and/or an N-methylamine group (NME) was added at the C-terminus with PyMOL version 1.5 (The PyMOL Molecular Graphics System, Schrödinger, LLC, http://www.pymol.org/). Titrating Asp, Glu and His residues were renamed to AS4, GL4 and HIP, respectively, to allow for their proper treatment as titratable groups within AMBER. After these preparation steps, missing hydrogens were added, disulfide bonds were defined and the topology file and the coordinate file were created with *tleap*. Finally, the *cpin* file was created with the *cpinutil.py* program from the Amber 12 molecular dynamics package. In the *cpin* file all possible titrating residues were defined as titrating.

### General setup and parameters of the MD simulation

The structure preparation was followed by two rounds of minimization, each comprising 2,500 steps of steepest descent followed by 2,500 steps of conjugate gradient minimization. In the first round, the hydrogen atoms were minimized while all heavy atoms were restrained with a constant force of 10 kcal/(mol∙Å^2^) to their initial positions. In the second round, no restrains were used to allow minimization of the entire system.

After minimization, the system was gradually heated from 10 K to 310 K within 0.1 ns while the protein was restrained with a constant force of 5 kcal/(mol∙Å^2^). The constant pH in the implicit solvent method was switched on and the solvent pH was set to a starting pH of either 7 or 8 for the protein simulations. The salt concentration was set to 0.1 M (based on Debye-Hückel), bonds involving hydrogen atoms were constrained with the SHAKE algorithm and a time step of 2 fs was used. Also the Langevin dynamics with a collision frequency of 2 ps^−1^ was used. The correctness of the protonation state was checked through Monte Carlo sampling of the Boltzmann distribution of protonation states as implemented in AMBER. In the AMBER setup the solution pH is given as an external parameter^8^ and the residues which were set as titrating can change their protonation state, while the protonation states for non-titrating residues are fixed. The different protonation states of those titrating residues were realized by different charge sets and the protonation state changes through the change of these partial charges on the atoms of the titrating residues^8^. Therefore, the titrating residues sample a Boltzmann distribution of protonation states using Monte Carlo and between the Monte Carlo steps the system is simulated with standard molecular dynamics^8^. In our simulation setup, the protonation check was performed at each time step of the molecular dynamics simulation. In the following equilibration phase (0.4 ns) the temperature was maintained at 310 K and the restraints were reduced to the alpha carbon atoms of the protein. All other parameters remained unchanged. In the production stage that was used for pK_a_ value calculation and analysis of protein dynamics, no restraints were used.

All molecular dynamics simulations were carried out with Amber12 or Amber14^32^ using the ff99SB^33^ force field. For all parts of the simulation the generalized Born solvent model igb = 2 and a cutoff of 30.0 Å were used for non-bonded interactions. In contrast to the simulations of proteins, the simulations of the model compounds were carried out at 300 K instead of 310 K. Intermolecular contacts and solvent accessibility were analyzed using the cpptraj^34^ module of Amber 12. Visualization was done with VMD 1.9.1^35^ and plots were created with gnuplot 4.6 (http://www.gnuplot.info/).

### Implementation of the pH titrating molecular dynamics (pHtMD) concept and pK_a_ value calculation

Our concept of pHtMD was implemented in the following fashion: Each protein simulation started at a neutral pH value and lasted for 1 ns. After each nanosecond at constant pH, the pH value of the solvent was slightly lowered or elevated and a further nanosecond was simulated using the final coordinates and velocities from the previous simulation round as an input. Using this procedure, a large number (>100) of consecutive 1-ns MD simulations were performed, slightly changing the solvent pH value in a defined direction, thus resembling a classical wet-lab titration experiment.

In order to determine appropriate rates for the pH change during the pHtMD, model calculations using four different rates were performed: 0.01 pH units/ns, 0.02 pH units/ns, 0.1 pH units/ns and 0.1 pH units/2 ns (data not shown). The rate of 0.02 pH units/ns was selected since it yielded a larger number of data points for pK_a_ value calculation compared to the faster rates, thereby facilitating the fitting procedure of the titration curves. The slower rate of 0.01 pH units/ns did not result in further improvement and was therefore not used due to its higher computational costs. A rate of 0.02 pH units/ns means that 50 ns of dynamics were calculated to achieve a pH change of one unit resulting in 100–300 ns of simulation time for the systems studied.

Each of the 100–300 pHtMD trajectories of 1 ns length was then used to calculate the pK_a_ values in the same fashion as described previously for independent CpHMD simulations that were performed for distinct solution pH values^11^. For each titratable group, the deprotonated fraction (f_deprot_) was used to calculate its pK_a_ value according to equation (1):


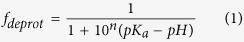


Fits according to this equation allow the estimation of pK_a_ values at the midpoint of titration and of the Hill coefficient n, which describes the cooperativity of various titrating groups^36^.

Post-processing of the f_deprot_ values was performed for buried side chains, which frequently exhibit a poor sampling of different protonation states: If the solvent accessible surface area (SASA) of a titratable side chain was <30% of the maximum SASA of this side chain, the respective data point was excluded from further analysis. Reference SASA values were deduced from the MD simulations of the Ace-X-Nme model compounds. The subsequent fitting procedure according to the equation above was then done only with the remaining data points.

To allow for a comparison with one commonly used pK_a_ predicting method, pK_a_ values of the side chains of aspartates, glutamates, and histidines were also predicted with PROPKA^37^,^38^ (http://propka.ki.ku.dk/).

## Additional Information

**How to cite this article**: Socher, E. and Sticht, H. Mimicking titration experiments with MD simulations: A protocol for the investigation of pH-dependent effects on proteins. *Sci. Rep.* **6**, 22523; doi: 10.1038/srep22523 (2016).

## Figures and Tables

**Figure 1:**
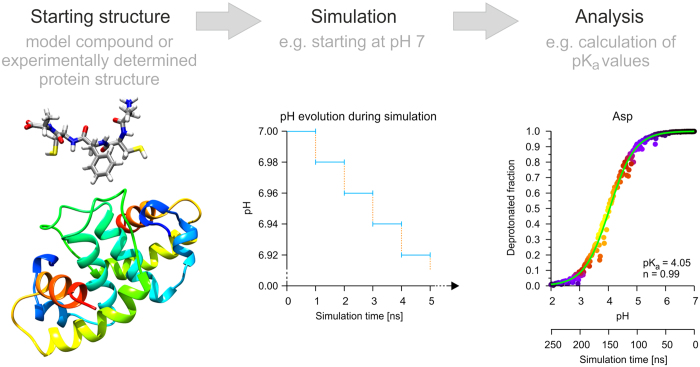
Workflow of pHtMD simulations. The pHtMD simulation starts with a model compound or an experimentally determined structure. At first, a 1 ns long CpHMD simulation is performed (blue line). The final coordinates and velocities are transferred (dashed orange lines) to serve as a starting point for the next 1 ns long CpHMD simulation (blue lines), which has now a slightly lowered pH compared to the previous 1 ns. These steps are repeated until the final pH value is reached. This example shows a systematic lowering of the pH; a systematic increase of the pH can be accomplished in an analogous fashion. The data obtained from the pHtMD can be analyzed with respect to different aspects, for instance pK_a_ values, conformational features or net charges of proteins.

**Table 1:** Predicted and experimental pK_a_ values of the SNase mutant Δ+PHS.

Residue	exp. pK_a_	pHtMD prediction^e^	CpHMD prediction	PROPKA prediction
Asp19	2.21	–a	4.1 (1.89)	4.22 (2.01)
Asp21	6.54	–a	–a	2.29 (4.25)
Asp40	3.87	–a	3.1 (0.77)	4.04 (0.17)
Asp77	<2.2	2.64 (>0.44)	3.6 (>1.40)	2.25 (>0.05)
Asp83	<2.2	–a	–b	2.72 (>0.52)
*Asp95*	2.16	3.23 (1.07)	3.6 (1.44)	2.69 (0.53)
*Glu10*	2.82	3.83 (1.01)	4.4 (1.58)	3.70 (0.88)
Glu43	4.32	4.03 (0.29)	–b	4.96 (0.64)
*Glu52*	3.93	3.77 (0.16)	4.3 (0.37)	3.87 (0.06)
*Glu57*	3.49	3.74 (0.25)	4.3 (0.81)	4.41 (0.92)
*Glu67*	3.76	3.84 (0.08)	4.39 (0.63)	3.61 (0.15)
*Glu73*	3.31	3.84 (0.53)	4.2 (0.89)	4.51 (1.20)
*Glu75*	3.26	4.16 (0.90)	4.0 (0.74)	3.65 (0.39)
*Glu101*	3.81	3.69 (0.12)	3.5 (0.31)	5.25 (1.44)
*Glu122*	3.89	3.27 (0.62)	3.8 (0.09)	3.83 (0.06)
*Glu129*	3.75	3.76 (0.01)	4.28 (0.53)	4.48 (0.73)
*Glu135*	3.76	3.58 (0.18)	4.2 (0.44)	3.27 (0.49)
His8	6.50	6.08 (0.42)	–c	6.29 (0.21)
His121	5.25	5.89 (0.64)	–c	6.43 (1.18)
sum of differences:^d^		**4.93**	**7.83**	**6.85**
average deviation:^d^		**0.45**	**0.71**	**0.62**

Experimental pK_a_ values for Asp/Glu and His residues were taken from the studies of Castañeda *et al.*^16^ and Fitch *et al.*^42^, respectively. The previous CpHMD study refers to data from Williams *et al.*^11^. Deviations from experiment are given in parentheses.

^a^No prediction possible.

^b^Value not considered here due to high STD (≥5) of the prediction.

^c^Not investigated.

^d^Calculated only for those residues (marked in italics) for which experimental pK_a_ values were available and predictions with all methods were possible.

^e^Values were averaged over six simulations.

**Table 2:** Predicted and experimental pK_a_ values of HdeA.

Residue	exp. pK_a_	pHtMD prediction^b^	pH-REMD prediction		PROPKA prediction
			dimer	monomer	
Glu19	4.38	4.07 (0.31)	4.1 (0.28)	4.3 (0.08)	4.77 (0.39)
Asp20	3.66	4.11 (0.45)	3.1 (0.56)	4.2 (0.54)	2.46 (1.20)
Asp25	3.71	2.75 (0.96)	3.1 (0.61)	3.8 (0.09)	2.82 (0.89)
Glu 26	4.57	4.37 (0.20)	4.0 (0.57)	4.5 (0.07)	4.74 (0.17)
Glu 37	–a	4.79	6.4	4.5	7.19
Asp 43	3.87	3.78 (0.09)	3.9 (0.03)	3.6 (0.27)	3.59 (0.28)
Glu 46	4.07	3.87 (0.20)	3.8 (0.27)	4.3 (0.23)	3.94 (0.13)
Asp 47	4.14	4.18 (0.04)	4.4 (0.26)	4.1 (0.04)	2.96 (1.18)
Asp 51	3.83	3.72 (0.11)	3.2 (0.63)	3.7 (0.13)	3.71 (0.12)
Asp 69	3.74	3.64 (0.10)	3.7 (0.04)	3.6 (0.14)	2.92 (0.82)
Asp 76	3.75	3.45 (0.30)	3.5 (0.25)	3.8 (0.05)	3.23 (0.52)
Glu 81	4.23	4.11 (0.12)	4.3 (0.07)	4.5 (0.27)	4.65 (0.42)
Asp 83	3.97	3.59 (0.38)	3.6 (0.37)	4.0 (0.03)	4.07 (0.1)
sum of differences:		**3.26**	**3.94**	**1.94**	**6.22**
average deviation:		**0.27**	**0.33**	**0.16**	**0.52**

Experimental pK_a_ values were taken from the study of Garrison & Crowhurst^22^. pH-REMD values refer to the study by Ahlstrom *et al.*^21^.

^a^pK_a_ value could not be determined experimentally.

^b^Averaged over both protein chains of dimeric HdeA.
