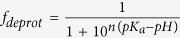# Erratum: Mimicking titration experiments with MD simulations: A protocol for the investigation of pH-dependent effects on proteins

**DOI:** 10.1038/srep25324

**Published:** 2016-05-03

**Authors:** Eileen Socher, Heinrich Sticht

Scientific Reports 6: Article number: 2252310.1038/srep22523; Published online: 03032016; Updated: 05032016

This Article contains errors in Equation 1.


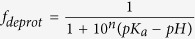


should read: